# The effect of oxidized low-density lipoprotein combined with adriamycin on the proliferation of Eca-109 cell line

**DOI:** 10.1186/1476-511X-10-108

**Published:** 2011-06-29

**Authors:** Hao Li, Qing D Li, Ping Zhi Wang, Mei Shu Wang, Jia Cui, Tao Yu Diao, Qing Hui Li

**Affiliations:** 1 Qilu Hospital, Shandong University, Jinan, P.R. China; 2 College of Public Health, Shandong University, Jinan, P.R. China; 3 Institutes of Basic Medicine, Shandong Academy of Medical Sciences Jinan, P.R. China

**Keywords:** Esophagueal squamous cell line, low-density lipoprotein, adriamycin, apoptosis, gene, protein, expression

## Abstract

**Background:**

The purpose of this study was to identify the affect on the proliferation Eca-109 cells treated with oxidized low-density lipoprotein (ox-LDL) combined with adriamycin (ADM).

**Methods:**

Eca-109 cell were cultured in the presence of oxLDL/ADM, and cell proliferation tested by MTT and cell apoptosis was monitored by the proportion of apoptosis and cell cycle by flow cytomester. We simultaneously evaluated the level of associated- apoptosis Bcl-2, Bax, and Caspase-3 gene mRNA and protein.

**Results:**

OxLDL were cytotoxic and activate apoptosis. OxLDL combined with ADM significant enhanced the proportion rate of apoptosis on a time and dose dependency. The expressions of the inhibiting apoptosis Bcl-2 gene mRNA and protein were down regulated, whereas, the expressions of the promoting apoptosis Bax, and Caspase-3 genes mRNA and protein were up regulation.

**Conclusion:**

These results suggested that oxLDL have cytotoxicity and activate apoptosis on the Eca-109 cells. OxLDL combined with ADM have a synergistic effect on the apoptosis induced Eca-109 cells. Furthermore, oxLDL may contribute to the improvement of clinical chemotherapy of cancer need to make further investigation.

## Background

### Nutrition therapy for patients with terminal cancer-associated cachexia

Weight lost and muscle wasting is a major characteristic of the cachexia associated with diverse pathologies such as terminal cancers, bacterial sepsis, and AIDS [1]. According to recent findings, the progressive weight loss and muscle wasting seen in cancer patients can be halted or even reversed by giving them a high-energy and high-protein supplement containing omega-3 fatty acids [2]. Various experimental and clinical studies have proved the benefit of consuming a diet supplemented with high-quality proteins (rich in essential amino acids) [3], omega-3 fatty acids [4,5], and antioxidants [6].

A current study reported that a crayfish enzymatic extract (a diet formulated), which characterized by its high contents in essential amino acids, omega-3 fatty acids and carotenoids, used for the treatment of cancer-associated cachexia in a rat model, and the results showed it can increase the survival of tumor-bearing animals and meliorate the cachexia symptoms - anorexia and body mass loss (muscle and adipose tissue) [7,8]. Recently a work reported the result of preoperative nutritional deficiency on mortality after radical cystectomy for bladder cancer ( nutritional deficiency, as measured by preoperative weight loss, body mass index and serum albumin), is a strong predictor of 90-day mortality and poor overall survival [9].

### Oxidized low- density lipoprotein (oxLDL) anti- tumor

Pathophysiologic effects of oxLDL in atherogenesis have been established. Recent studies show an association with tumorigenesis [10]. Oxidative stress is a term used to denote the imbalance between the concentrations of reactive oxygen (ROS) and nitrogen (RNS) species and the antioxidative defense mechanisms of the body [11]. ROS cause oxidation of lipids, proteins, and DNA *in vivo *[12,13]. LDL is susceptible to oxidation, resulting in the formation of oxLDL [14,15]. In recent years, there have been many studies suggesting that excessive lipid peroxidation may play a key role in cancer development [16] Furthermore, it has been found in animal models that a high level of lipid peroxidation is closely associated with carcinogenesis [17,18].

Our previous study showed that oxLDL was decreased in patients with esophageal squamous cell carcinoma (ESCC). The decreasing oxLDL negatively relate to the different stages of the development of esophageal squamous cell carcinoma, which starts from normal epithelium to basal cell hyperplasia, dysplasia or carcinoma in situ and, finally, to invasive ESCC [19-21].

Given the results of these previous studies, we hypothesize that if patients with cancer were given nutrition therapy, especially omega-3 fatty acids, in early stage, the terminal cancer-associated cachexia would delay occurs. The fact prompts us, a composition of lipids, such as oxLDL, may be associated with tumor progression. The aim of the present study was to investigate the role of oxLDL and oxLDL combined with ADM (ADM) in Eca-109 cell apoptosis. Through their pro-apoptotic effect observed *in vitro*, oxLDL would enhance the efficiency of inhibiting the proliferation of cancer cells treated by ADM and thus might be considered as nutritional indicator in the progression of ESCC.

## Methods

### Cell culture

Human ESCC cells, Eca-109, was supplied by the Affiliated Hospital of Tumor Treatment and Prevention of Shandong Academy of Medical Sciences repository and cultured in RPMI1640 medium (Gibco, Los Angeles, CA, USA) supplemented with 10% heat-inactivated new-born calf serum (HangZhou Sijiqing Biological Engineering Materials Co. Ltd. China), and cells were grown in a humidified incubator at 37°Cand 5%CO_2_.

### Assay of in vitro oxLDL and ADM sensitivity

An enzyme-linked immunosorbent assay (ELISA) kits of oxLDL purchased from ADL (Adliteram Diagnostic Laboratories Lnc., USA). The 3-(4, 5-dimethylthiazol-2-yl)-2, 5-diphenyltetrazoliumbromide (MTT) assay [22] was tested to calculate the inhibition rate of cell viability treated by drugs. Briefly, the untreated Eca-109 cells were generally in exponential growth phase at the end of the 7-day incubation. All the results represented the average of a minimum of 6 wells. The Eca-10^9^ cells were grown on a 96-well plate at 1 × 105 cells/ml in complete RPMI 1640 medium.

Oxidized LDL and ADM were added at various different drug concentrations. Eca-109 cells without drugs in medium were used as the blank controls. After the drugs treatment for 20 h, 44 h, and 68 h at 37°C, 20 μl MTT were added into each well and incubated for a further 4 h respectively, then the medium were removed and 150 μl dimethyl sulfoxide were added to each well to dissolve the formazan crystals. Absorbance values were measured with spectrophotometer at the wavelength of 570 nm.

The number of cells was also established using a hemocytometer and the cell viability was determined by the trypan blue exclusion test. The inhibition rate of cell viability was calculated using the following formula: Inhibition of cell viability = (1-average a value of experimental group/average a value of control group) × 100%.

### Assay of in vitro oxLDL combined with ADM sensitivity

Eca-109 cells were treated with 40 μg/ml oxLDL and 0.1 μg/ml ADM for 48 h. Cells were harvested by centrifugation, and then were tested by MTT to calculate the inhibition rate as above description.

### Determination of Eca-109 cell Apoptosis

#### Cell apoptosis assay by AnnexinV/PI

Twenty-four hours, 48 h and 72 h, after culture, cells were trypsinized, counted1 × 10^6^ cells per sample were washed twice by ice-cold PBS solution, and then 5 μl of annexin V-FITC (AV) and 10 μl of propidium iodide (PI) (Sigma) were added to 100 μl of cell suspension, followed by incubation for 15 min at room temperature in the dark. Finally, 400 μl of binding buffer was added to each sample, and filtered by 300-mesh nylon net before EPICS XL (Beckman Coulter, Bren CA USA) flow cytometer.EXPO32 ADC Analysis Software was used to analyze the data.

#### Cell cycle analysis by flow Cytometry

Twenty-four hours, 48 h and 72 h, after culture, cells were trypsinized, counted1 × 10^6^ cells per sample were washed twice by ice-cold PBS solution, and then fixed with 70% ice-cold ethanol overnight at 4°C. Fixed cells were washed twice with PBS and fixed with 70% ice-cold ethanol overnight at 4°C. Fixed cells were washed twice with PBS. Cellular DNA was stained with 500 μl propidium iodide solution (0.005%PI, 0.1%Triton X-100, 0.1 mM EDTA,0.01%Rnase,0.1 ml PBS), followed by incubation for 30 min at 4°C in the dark, and filtered by 300-mesh nylon net. Samples were analyzed on an EPICS XL flow cytometer (Beckman Coulter, Bren CA USA). The percentage of cells in each phase of the cell cycle was estimated.

### Semiquantitative RT-PCR assay

*In vitro *total mRNA was extracted from the cells with Trizol reagent (Invitrogen Co. California, USA) according to manufacturer's instructions. Single stranded cDNA was synthesized by reverse transcription from 1 μg of total RNA using Reverse Transcriptase RNAse M-MLV (Invitrogen Co. California, USA) and oligo-dT. The amplification was performed in a final volume of 50 μl, containing 5 μl cDNA, 0.5 μl of each oligonucleotide primer, 1 μl of each dNTPs, and 1 units of Taq DNA polymerase. Amplification was carried out in a Thermal Cycler. All oligonucleotide primer sets used to measure the expression levels of Bcl-1, Bax, Caspase-3 gene mRNA, were previously described [11,17,23]. The primers used for measurements of the mRNA expression levels of genes (upstream and downstream, respectively) were CGACGACTTCTCCCGCCGCTACCGC and CCGCATGCTGGGGCCGTACAGTTCC for Bcl-2 mRNA; TCCACCAAGAAGCTGAGCGAG and GTCCAGCCCATGATGGTTCT for Bax mRNA; CCCATTTCTCCATACGCACT and TGACAGCCAGTGAGACTTGG for Caspase-3 mRNA; GTGGGGCGCCCCAGGCACC and CTCCTTAATGTCACGCACGATTTC for β-actin serves as loading control. Circulating condition: 94°C one min, 58°C one min, 72°C one min, thirty cycles, then 72°C prolong 7 min. End products were identified by electrophoresis using 1.5% agarose gel.

### Western blot analysis

Cells treated for 48 h as described above were washed twice in ice-cold PBS and protein extracts of Eca-109 cells were prepared by lysis buffer (150 mM NaCl, 1%NP-40, 0.5% 1nM Sodium orthorandate, 0.1% sodium dodecyl sulfate (SDS), 50 mM Tris HCl, 10 mM EDTA, and 1 mM PMSF) for 30 min at 4°C. Samples were next centrifuged for 15 min at 10,000 g. Protein concentrations of supernatants were determined. For each sample, 60 μg of protein was loaded on a 12.5% SDS-polyacrylamide gel, electrophoresed, and transferred to a nitrocellulose membrane (Protran; Schleicher and Schuell, Florham Park, NJ). Each membrane was blocked for 1 h at room temperature with blocking buffer (TBS containing 0.1% Tween 20 and 5% milk powder). Primary antibodies (applied for 1 h at room temperature, or overnight at 4°C) were: anti-Bcl-2 (Santa Cruz Biotechnology, Inc., Heidelberg, Germany), anti-Bax (Santa Cruz), and anti-actin (mouse monoclonal C-2, Santa Cruz). Antibodies were diluted, thereafter, membranes were incubated for 1 h with HRP-labeled secondary antibodies (Amersham Pharmacia Biotech, Uppsala, Sweden), goat anti-rabbit, and the blots were finally developed using an ECL Imaging Densitometer (Amersham Bioscience, Buckinghamshire, UK).

### Microscopy

Morphological changes in cancer cells treated with oxLDL and ADM were observed by the trypan blue exclusion test using an Olympus microscope. During the procedure, cell morphology was observed under light microscope for different time.

### Statistical Analyses

Statistical calculations were carried out with the SPSS 15.0 for Windows software package. Results are expressed as the mean ± standard deviation of independent experiments. Statistical differences between two groups were calculated by the unpaired Student's *t*-test; and the differences among more than two groups were tested by one-way ANOVA or two-way ANOVA, and follow the Bonferroni test for sub- two groups comparison, *P *values < 0.05 were considered to be significant.

## Results

### OxLDL have cytotoxic effects on Eca 109 cell in vitro

**Figure 1:**
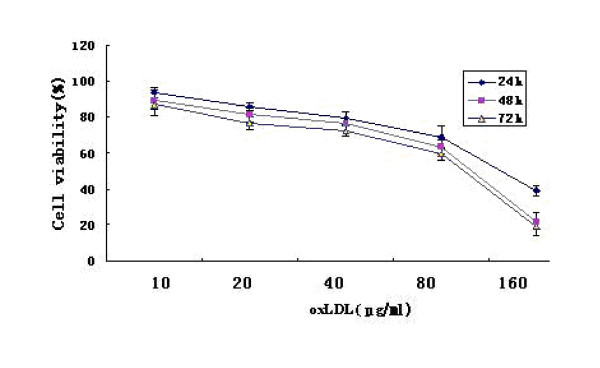
**The viability of Eca-109 cells treated with oxLDL in the MTT test**.

The effect of oxLDL was examined in Eca 109 cell line. OxLDL decreased cell viability and proliferation in a dose- and time- dependent manner in the test (figure 1).

### OxLDL induces apoptosis

**Figure 2:**
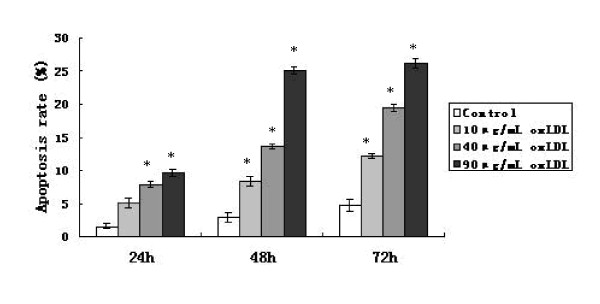
**The frequencies of apoptosis for Eca-109 cells treated with three concentration (10, 40, 90 μg/ml) of oxLDL observed after 24 h, 48 h and 72 h of the treatment**. *: Compared with the control, p < 0.05.

The apoptotic frequencies of Eca-109 cells treated with three concentrations (10, 40, 90 μg/ml) of oxLDL increased in a dose- and time- dependent manner in the flow cytometer test (figure 2).

### Assay of in vitro oxLDL combined with ADM sensitivity

**Figure 3:**
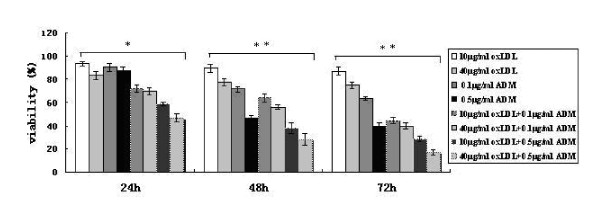
**The frequencies of viability for the Eca-109 cell treated with oxLDL, ADM on alone or their combination**. *: p < 0.05, **: p < 0.01 in ANOVA test.

Compared with the viability rates of Eca-109 cell treated by oxLDL or ADM alone, the viability rates of Eca-109 cells treated by oxLDL combined with ADM also decreased along with a dose- and time- dependence. The viability rates of Eca-109 cell treated by 40 μg/ml oxLDL combined with 0.5 μg/ml ADM were significantly lower than that in all other groups at 24 h, 48 h, and 72 h after the treatment in the MTT test (figure 3).

### Apoptotic morphology

**Figure 4:**
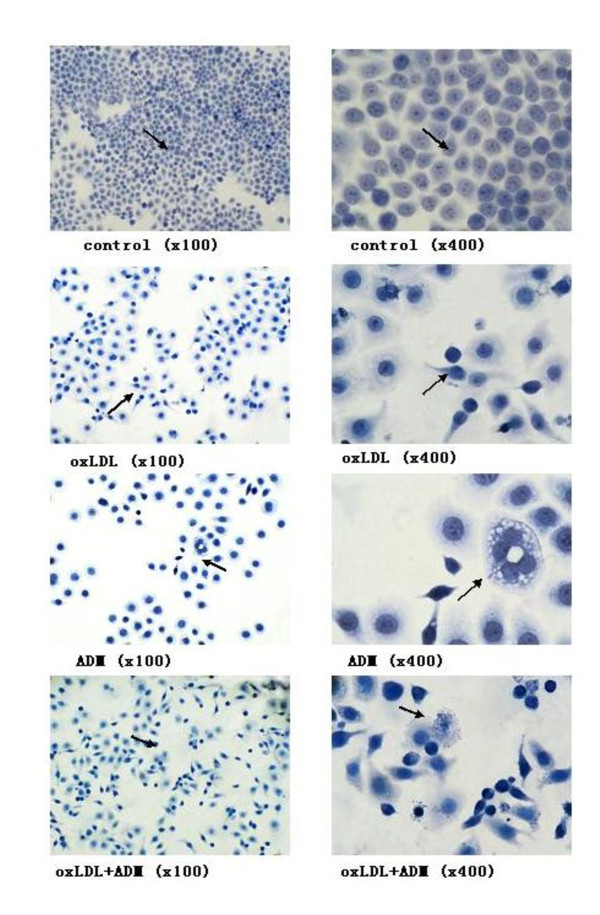
**Morphology of Eca-109 cell apoptosis in light microscopy (magnification with x100 and x400, respectively for the same slice)**; When the Eca-109 cell treated with the drugs 48 h after the treatment, the morphology of the cell showed nuclear chromatin condensation and fragmentation, plasmic budding, phagocytosis of the extruded and apoptotic body. Note: The doses of drugs treated with 40 μg/ml oxLDL, 0.1 μg/ml ADM, and both of them combined.

Typical apoptosis morphological changes were found in Eca-109 cell line 48 h after treatment with 40 μg/ml oxLDL, 0.1 μg/ml ADM and both of them combined. The changes included nuclear chromatin condensation and fragmentation, plasmic budding, phagocytosis of the extruded and apoptotic body (figure 4).

### OxLDL combined with ADM induce the apoptosis and the changes of cell cycle

**Figure 5:**
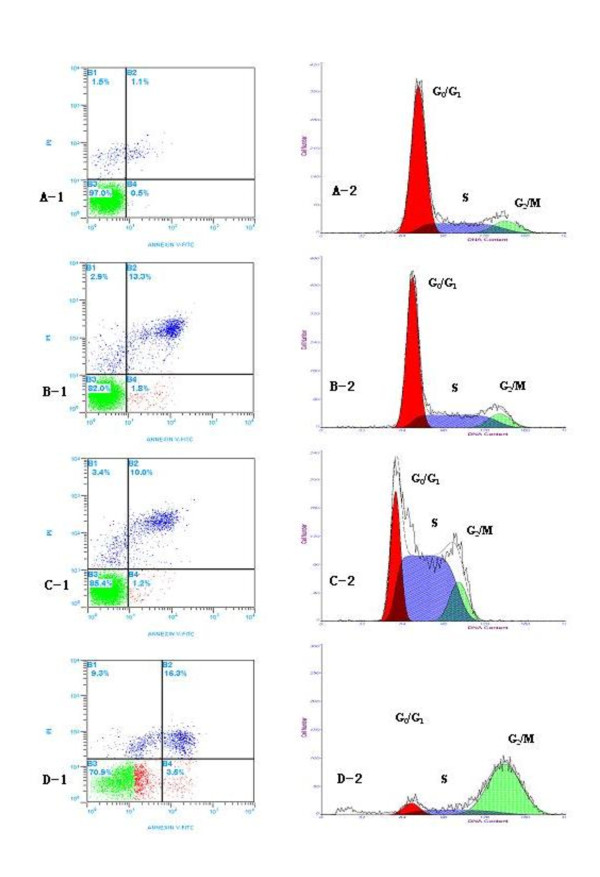
**A-1, B-1, C-1 and D-1 showed the distribution of percentages of apoptosis;, and A-2, B-2, C-2 and D-2 showed the distribution of percentages of cell cycle for Eca-109 cells treated with oxLDL or ADM alone and both of them combined after 48 h of the treatment**. **For cell cycle: A-2**: 67.37 ± 5.49%, 22.03 ± 0.73% and 10.60 ± 0.26% in the G1/G0, S and G2/M phase of control; **B-2**: 59.45 ± 4.53%, 29.86 ± 1.77% and 10.69 ± 0.62% in the G1/G0, S and G2/M phase of the oxLDL; **C-2**: 24.12 ± 3.64%, 62.59 ± 4.29% and 13.28 ± 1.45% in the G1/G0, S and G2/M phase of the ADM; **D-2**: 8.73 ± 2.48%, 15.61 ± 5.17% and 75.66 ± 3.64% in the G1/G0, S and G2/M phase of the above oxLDL and ADM combined, respectively. The proportions of apoptosis and phase of cell cycle in 40 μg/ml oxLDL + 0.1 μg/ml ADM group were significant higher than that in other groups, p < 0.05. **Dose**: **A-1 **and **A-2**: control group; **B-1 **and **B-2**: 40 μg/ml oxLDL; **C-1**and **C-2**: 0.1 μg/ml ADM; **D-1 **and **D-2**: 40 μg/ml oxLDL + 0.1 μg/ml ADM. Abbreviation: ADM, adriamycin.

Compared with the apoptotic frequency of Eca-109 cell treated by 40 μg/ml oxLDL or 0.1 μg/ml ADM alone, the apoptotic rate of Eca-109 cells treated by 40 μg/ml oxLDL combined with 0.1 μg/ml ADM was significantly higher in the flow cytometer test (figure 5).

The abilities of 40 μg/ml oxLDL or 0.1 μg/ml ADM on alone or their combination to stimulate the Eca-109 cells into the cell cycle were analyzed by flow cytometry. Cells treated with 40 μg/ml oxLDL or 0.1 μg/ml ADM had substantial increases in the proportion of cells in S phase over time. For example, after treatment with 40 μg/ml oxLDL, 29.86% of cells were in S at 48 h. After treatment with 0.1 μg/ml ADM, 62.59% of cells were in S phase at 48 h. In contrast, after treatment with both of them combination, 75.66% of cells was in G2/M blockage at 48 h (figure 5).

### RT-PCR the expression of Bcl-2, Bax and caspase-3 genes

**Figure 6:**
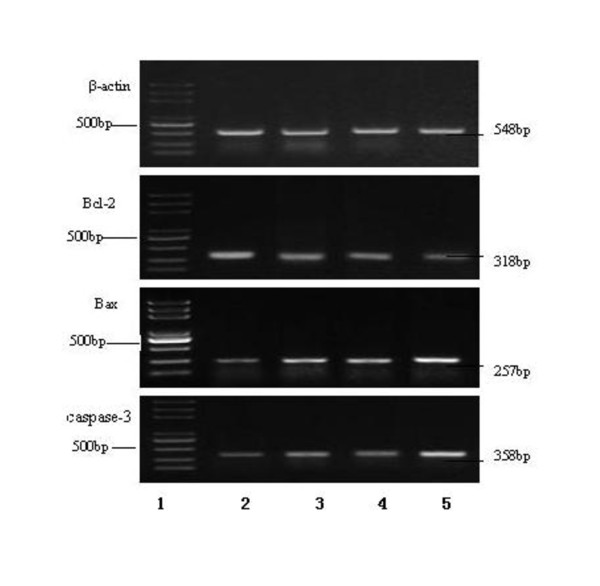
**The RT-PCR expression of Bcl-2, Bax and Caspase-3 gene mRNA for the Eca-109 cell treated by the oxLDL, ADM, and both of them combined for 48 hours**. β-actin serves as loading control. Note: line 1: DNA marker; line 2: control; line 3: 40 μg/ml oxLDL; line 4: 0.1 μg/ml ADM; Line 5: 40 μg/ml oxLDL + 0.1 μg/ml ADM. Abbreviation: ADM, adriamycin.

Forty eight hours After the Eca-109 cells treated with oxLDL, ADM or their combination, the levels of Bcl-2 gene expression were down-regulated, while the levels of Bax and caspase-3 genes expression were up-regulated, compared with that in the control group (figure 6).

### Protein expression of Bcl-2, Bax and caspase-3 genes in West blot test

**Figure 7:**
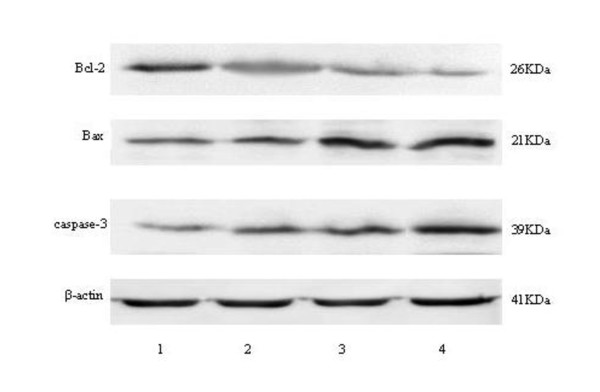
**The protein expression of Bcl-2, Bax and Caspase-3 genes for the Eca-109 cell treated with the oxLDL, ADM, and their combination for 48 h**. β-actin serves as loading control. Note: line 1: control; line 2: 40 μg/ml oxLDL; line 3: 0.1 μg/ml ADM; Line 4: 40 μg/ml oxLDL + 0.1 μg/ml ADM. Abbreviation: ADM, adriamycin.

After 48 h of the Eca-109 cells treated with oxLDL, ADM or their combination, the levels of protein expression of Bcl-2 gene decreased, while the levels protein expression of Bax and caspase-3 genes increased, compared with that in the control group (figure 7).

## Discussion

It is well established that lipid-lipoprotein particles among density classes are metabolically processed forming a sequence of diminishing size and lipid distribution, beginning with LPL activity on triglyceride in chylomicron and continuing through HDL, resulting in increasing density and loss of lipid due to enzymic activities during the course of normal lipid transport [24]. Tumor burden also resulted in a marked reduction in adipose tissue, together with a clear hyperlipemia. The loss of fat mass is the result of two altered processes: first, an increase in lipolytic activity, which results in a significant release of both glycerol and fatty acids [25], and second, a marked decrease in the activity of lipoprotein lipase (LPL). This enzyme is responsible for the cleavage of both endogenous and exogenous triacylglycerols to form glycerol and fatty acids, which is reflected as hypertriglyceridemia [26].

Pathophysiologic effects of oxLDL in atherogenesis have been established. Recent studies show a positive correlation between increased serum oxLDL levels and an increased risk of colon, breast and ovarian cancer [27-29]. An earlier study reported that lipids might primarily affect the gonads, and subsequently higher estradiol secretion could influence the development of malignancies in these sites [30]. Later study showed that it is possible that LDL, which is susceptible to oxidation, result in high lipid peroxidation contributing to carcinogenesis [27].

In the past years, there has been a growing body of evidence that excessive lipid peroxidation may play a key role in cancer development [31,32] and in animal model associated with carcinogenesis [17,33]. Lipid peroxidation metabolites damage DNA and can seriously inhibit DNA repair capacity through their direct interaction with repair proteins [34]. Circulating oxLDL is also quite reliable biomarker of lipid peroxidation [17]. OxLDL have been reported as a potent independent mitogenic factor [35] that induces proliferation or cell death [36] and furthermore, could contribute to the release of cytokines and growth factors-associated with cancer [37]. However, a meta-analysis on patients suffering from various diseases revealed that only under severe pathological conditions, e.g. HIV infection, all the indices of oxidative stress correlate with each other [38].

The present investigation, for the first time, identified cytotoxicity on the proliferation of Eca-109 cell line and showed time and dose dependency. The apoptosis of Eca-109 cells treated by oxLDL also showed in time- and dose- manner. The RT-PCR expression of Bcl-2, Bax and caspase-3 genes mRNA and protein expression of them confirmed the mechanism of the inducing apoptosis by oxLDL. This fact suggests that the physiological amount of oxLDL involves in the process of cell apoptosis, clears the mutation cells or controls the proliferation of cancer cells in vivo. At the advanced stage of cancer, the activity of lipoprotein lipase in vivo decreases, and in consequence, the level of oxLDL declines, promoting an uncontrolled proliferation of tumor cell. Meanwhile, the activity of lipolysis increases, leading to occurrences of the cancer-associated cachexia.

It is confirmed that non-oxidized low-density lipoprotein (nLDL) has no effect on cell lines viability and proliferation [35]. OxLDL with cytotoxicity or autophagy dependences cell types. Under some conditions, oxLDL can be cytotoxic. OxLDL can induce changes in cell cycle protein distribution and expression characteristic of a controlled, adaptive response to a chronic pathological condition. Autophagy is another form of programmed cell death mediated by the lectin-like oxLDL receptor-1(LOX-1)-dependent [39]. Previous studies showed that oxLDL (more than 10 μg/ml) has cytotoxic effects on cancer cells in vitro and activate apoptosis and autophagy. OxLDL, applied at physiologic concentrations, decreased cell viability and proliferation in a dose-dependent manner in cell lines tested such as HT29 (colon), OVCAR3 (ovarian), HeLa (cervical), MCF7 (breast), A549 (lung), and PC3 (prostate) [40]. However, when the quiescent human fibroblasts and rabbit smooth muscle cells (VSMC) were treated with oxLDL at physiological ranges (0, 10, or 50 μg/ml) at 24-48 h, the total cell number of them significantly increased [35].

Now in another test, we also observed, the human umbilical vein endothelial cells (HUVEC) was treated with the dose lass than 40 μg/ml oxLDL at physiological ranges for 24-48 h. The resulted in significant increases in total HVEC cell counts at both time points. However, when the cell treated with larger than 40 μg/ml oxLDL, the inhibiting proliferation phenomenon observed at the same time points for the cells (data not shown).

More than two decades ago, epidemiological studies showed a U-shaped relationship between total cholesterol (TC) levels and risk of all-cause mortality. The relationship between the baseline serum cholesterol level to total mortality was attributed to the high number of deaths associated with serum cholesterol level at the high end of the distribution (mainly due to coronary heart disease) and at the low end (mainly due to cancer) [41-43]. Recent study reported that in atherosclerosis, ox-LDL linkage with its receptor LOX-1 activates the inflammatory pathway through NF-κB, leading to cell transformation. LOX-1 is important for maintaining the transformed state in developmentally diverse cancer cell lines and for tumor growth, suggesting a molecular connection between atherogenesis and tumorigenesis [10]. One study reported that low dose oxLDL has bilateral adjustment characteristics on the proliferation of quiescent human fibroblasts and rabbit smooth muscle cells. Western blot analysis revealed that oxLDL-stimulated cell proliferation was associated with significant increases in the expression of proteins that regulate entry into and progression through the cell cycle. Surprisingly, the expression of cell cycle inhibitors (p21 and p27) was stimulated by oxLDL as well, but this was to a lesser extent than the effects on cell cycle-activating proteins [36]. In the present study, we also found, the Eca-109 cells were treated with more than 40 μg/ml oxLDL the cytotoxicity of it was significantly increase on a time- and dose- dependency. It is possible that the levels of oxLDL in vivo are dynamical changes based on the body's conditions. Under some condition, oxLDL can be cytotoxic.

One clinical study indicated that the levels of oxLDL were increased among both breast and ovarian cancer patients as compared to the control subjects [44]. Another previous study showed that fasting lipid and lipoprotein studies on 38 consecutively diagnosed children with acute lymphoblastic leukemia (ALL). The results showed that the level of LDL decreased in the process of chemotherapy of the malignant tumor and recovered to normal level after the treatment. For those patients with ALL who had a long-term survival, the normal levels of LDL also were important [45]. The effects of tamoxifen treated the patients with breast cancer showed that LDL particle diameter correlated negatively with plasma triglyceride (TG) (r = -0.62; p < 0.001). Tamoxifen-induced fatty liver in breast cancer patients may be atherogenic, via increased TG and consequent small, easily oxidized LDL particles [46]. The smaller diameter of LDL particles, the more easily oxidized into oxLDL.

Recent review the results of cancer-related malnutrition patients with and without nutritional supplements showed the weight loss is multifactorial but can be generally separated into two components. One component included those outcomes that are the result of the metabolic abnormalities as a direct consequence of the tumor, and a second component including results of treatments, psychological issues, and others. For people with cancer the implement nutrition interventions are effective, but there is no clarity in best options for nutrition management [47]. More clinical observation of the dynamic changes of oxLDL for other type cancer in chemotherapy need to be confirm in future.

Another major finding of the present study is a synergistic effect on inducing apoptosis of Eca-109 cells treated by the combination of 40 μg/ml oxLDL and 0.1 μg/ml ADM. The proportion rate of apoptosis ECA-109 cells treated with their combination significantly increased (19.79 ± 1.32), compared with that 40 μg/ml oxLDL (15.11 ± 0.61), 0.1 μg/ml ADM (11.27 ± 0.54). As well known, the chemical-therapy drugs of cancer, such as ADM, have toxicity, if oxLDL combined with the cytotoxic drugs could use in the clinic, may be more benefit for the patient's therapy. Further studies are required in order to elucidate whether oxLDL play a causative or merely consequential role in cancer process and to designate a novel approach in the combination therapeutic strategies.

## Conclusion

These results suggested that oxLDL have cytotoxicity on the proliferation of Eca-109 cell line and showed time and dose dependency. OxLDL combined with ADM have a synergistic effect on the apoptosis induced Eca-109 cells. Furthermore, oxLDL may contribute to the improvement of clinical chemotherapy of cancer need to make further investigation.

## Abbreviations

oxLDL: oxidized low- density lipoprotein; ELISA: enzyme-linked immunosorbent assay; MTT: 3-(4, 5-dimethylthiazol-2-yl)-2, 5-diphenyltetrazoliumbromide; ESCC: esophageal squamous cell carcinoma; ADM: adriamycin.

## Authors' contributions

Members listed below made their respective contributions to this manuscript. Professor HL, QDL, PZW, MSW and JC designed the skeleton of this study, performed cell proliferation and apoptosis, expression of gene and protein and drafted the manuscript (they made the same contribution to this work). TYD and QHL carried out the data analysis and checked the manuscript. All authors read and approved the final manuscript.

## Competing interests

The authors declare that they have no competing interests.
